# Natural language processing to enhance rheumatoid arthritis care in clinical studies: a scoping review of applications, data, approaches, challenges and future directions

**DOI:** 10.1007/s00296-026-06195-0

**Published:** 2026-06-22

**Authors:** Yinan Huang, Sandeep K. Agarwal

**Affiliations:** 1 Department of Pharmacy Administration, School of Pharmacy, The University of Mississippi, Oxford, MS USA; 2 Department of Medicine Immunology, Allergy and Rheumatology, Baylor College of Medicine, Houston, TX USA

**Keywords:** Rheumatoid arthritis, Natural language processing, Clinical applications, Real-world evidence, Disease phenotyping, Social media analyses

## Abstract

Structured claims or EMR datasets have limitations, such as lacking important clinical variables, upcoding, or potential coding errors. Unstructured data powered with natural language processing (NLP) might bridge these gaps. We assessed the integration of NLP with unstructured data in advancing care for rheumatoid arthritis (RA). We conducted a scoping literature review search in PubMed, Embase, Web of Science and Directory of Open Access Journal to identify full-text studies published through February 27 2026. We used the search terms and relevant MeSH terms involving "rheumatoid arthritis" and "natural language processing” to identify English publishing studies that used the NLP methods while also considering any real world clincial application. Abstracts, reviews, reports, or commentaries were excluded. We extracted clinical problems, data, use of NLP and main findings across each study. Through the search terms, there identified a total of unique 345 citations after removing 54 duplicates. After assessing eligibility criteria, 27 studies met the inclusion criteria. We thoroughly reviewed all 27 qualified publications, and found that they were conducted in US, Japan, Germany, UK, and others. The tasks involved in these eligible studies were divided into several categories to show how NLP was explored to advance RA care: (1) extract a variety of clinical features to support predictive tasks; (2) identify cohorts of RA patients with specific phenotypes; (3) aid in ICD diagnosis codes to improve the precise diagnosis of RA or RA related comorbidities through extraction of clinical notes; (4) social media analysis; (5) extract information about RA-related medication use, indications, reasons for adjustment, and detecting medication induced safety signals. The unstructured datasets leveraged using NLP were divided into categories: medical health records, physician chart notes, purely social media texts, medical text notes and others. NLP, which is able to extract information from unstructured text data, has been increasingly used in clinical studies for RA. Recent work have shown that wide use of NLP to capture concepts from the unstructured clinical notes from EHR data, to improve the identification of RA or RA related comorbidities or RA disease phenotyping. Integrating NLP into structured data to detect observed confounders, in powering pharmacoepidemiologic studies of comparative effectiveness research in RA could be an important future area.

**Supplementary Information:**

The online version contains supplementary material available at 10.1007/s00296-026-06195-0.

## Introduction

Rheumatoid arthritis (RA) is a progressive autoimmune disease characterized by chronic inflammation of the synovial lining of peripheral joints, manifesting as pain and stiffness, which if left untreated could result in progressive damage to the affected joints [1]. The population of population with RA in the United States has reached more than 1.5 million, but is expected to increase in the coming years amid the trend of aging phenomenon [2]. It is associated with growing burden of economic and medical burden on the patients affected by this disease. By analyzing the US national survey data between 2016 and 2018, Ding et al. reported that the cost of RA care can be as high as $11.4 billion by considering total medical expenditures, out of pocket costs for use of prescription biologics and other RA care related cost; meanwhile, other evidence also showed that RA patients suffer from both deteriorated reduced health-related quality of life including physical, mental and cognitive components [3–5].

Artificial intelligence (AI), offers an innovative tool to leverage computational intelligence to perform tasks through learning patterns from underlying data. AI is able to perform a variety of tasks which typically need the source of human intelligence, such as natural language, perception, as well as other problem-solving. As a subset of AI, machine learning (ML) is a type of data-driven methods. Amid the wealth of extensive data in the healthcare, impact of the AI and ML in health science research and medicine is increasing, with different algorithms developed for the diagnosis, prognosis and other clinical task prediction across various disease areas [6]. As such, RA-related problems have also gradually attracted attention from researchers in the relevant field of AI, and in recent years, there observes a wider application of ML in various aspects of rheumatology, including disease detection, patient management and education, including early diagnosis and disease activity prediction, treatment response prediction and personalized medicine, flare prediction and monitoring [7–9].

While significant progress has been made in leveraging AI and ML around improving care for RA through using large databases (i.e., electronic health records [EHR] or administrative claims), gaps still remain [10, 11]. Real-world studies based on analyzing such large secondary datasets contain valuable patients’ health information recorded in the delivery of routine care might be effective in deriving evidence guiding clinical decision-making for RA, however readily available structured data are made for billing or collected through care delivery process, and often have limitations. For example, in relation to claims data, AI and ML are limited in the ability to capture key clinical confounders or outcomes of interests, such as RA disease activity, laboratory values and vital signs [12]. For another example, for electronic medical record (EMR) data, these models may have problems of missingness in some relevant clinical confounders, and also have limited generalizability due to only capturing the information within 1 provider system. Insurance claims data also have other known limitations including under-coding and errors in reporting of diagnosis coding [13]. As such, while structured secondary datasets have increasingly become an important tool to generate real-world observational research evidence, to advance clinical research in RA, challenges of using solely using these structured secondary datasets still exist [14]. In particular, one challenge specifically related to the ability to the identification of RA is that RA is a condition characterized by diverse disease symptoms, hence, identification and classification of RA events is difficult because of heterogeneity in clinical symptoms and phenotypes underlying RA difficulty to accurate diagnosis of RA because rheumatological conditions often involving multiple organs and presenting with heterogenous symptoms, making accurate diagnosis challenging simply based on analysis of structured data. Furthermore, structured dataset systems do not have accurate recording of prognostic clinical information to enable target-to-treat principle, and thus while clinical guidelines recommend the use of advanced disease-modifying antirheumatic drugs based on prognostic factors, the definition of such factors are usually not available in structured dataset. Overall, it underscores challenges to many RA-related problems in epidemiology and outcome research.

With the AI and ML growing, efforts to expand clinical inputs and outputs through mining the unstructured data are underway. NLP, as a more specialized subset area of AI and ML, referring to understand, process and analyze human natural language, allows extraction and detection of textual based information, from unstructured data [15]. NLP technique has been used to apply to extend features in analyzing unstructured clinical notes in the field of pharmacoepidemiology, the detection of medication related adverse drug events and healthcare research [16–18]. Specific for RA research, NLPs have been gradually applied in the rheumatology field, including systematically extracting disease related diagnoses from physician notes, yielding ascertainment of disease related clinically relevant characteristics or disease related outcome measures from unstructured data aiding EHRs [19–21]. For example, Humbert-Droz et al. demonstrated the use of NLP in extracting RA related outcome measures from rheumatology physician notes within the ACR’s RA RISE registry dataset, highlighting the potential of NLP in addressing some of the most challenging tasks in the field [19]. Overall, this novel NLP method has the potential to leverage information from unstructured dataset, such as clinical textual notes from EMR or physician chart reviews or other unstructured text-based data might provide additional information.

Nevertheless, in the era of adopting precision medicine for RA, some existing reviews have synthesized current applications and knowledge in the use of broad AI and ML approaches in RA, such as risk prediction modelling, diagnosis tool, disease management, evaluate treatment responses for RA. While growth of unstructured data context may drive the further direction, synthesis of current NLP use in RA is important, existing reviews in this particular interdisciplinary emerging area specifically for RA care are limited. In particular, current systematic reviews have evaluated the use of NLP techniques in diagnosis and prediction of cognitive impairment, infectious diseases, mental health intervention, cancer phenotyping, general medicine [22–26]. To our best knowledge, the integration of ML has only emerged recently and, only 3 reviews have involved the use of NLP to evaluate RA care relevant clinical studies [27–29]. Of them, Tariq et al. and Stafford et al. broadly outlined current applications of AI use for diagnosis, disease management, treatment responses and clinical prognosis to improve care for RA [27, 28]. Omar et al. specifically summarized the NLP use in rheumatology until 2024, but it focused on the broad disease of rheumatology, involving RA, SpAs, gout and other rheumatic conditions, without focused evaluation of NLP in RA [29]. Thus, the performance of NLP technique applied in large-scale, real-world clinical problems specifically for RA has not been systematically evaluated. The possible applications of integrating NLP techniques in extending features outside of structured EHR data at the population level observational research remains unclear. Therefore, it underscores the need for a comprehensive review to consolidate interdisciplinary progress and direct future efforts. In response to this identified need, this scoping review aimed to provide a comprehensive overview of various applications of NLP in RA care, with a particular focus on available data, modelling, and evaluation metrics in NLP.

The review aimed to summarize the current application of NLP for clinical research addressing the needs for patients with RA, with respects to extracting information to enhance RA related pharmacoepidemiologic research or RA disease related outcome research. Specifically, this scoping review especially focused on three key aspects: clinical problems, datasets used and features considered. Given the growing volume and potential value of unstructured data in RA care, findings of this scoping review are of clinical implications and methodology relevance, because such finding might be translated to support the measurement of outcome measures, identification of important clinical confounding variables towards improving RA-related clinical research and quality initiatives.

## Methods

A scoping review was conducted by following the Preferred Reporting Items for Systematic Reviews and Meta-Analysis Extension for Scoping Reviews (PRISMA-ScR) [30]. As per PRISMA flow diagram, there includes three key steps: search identification, study screening and inclusion. Study protocol is available in supplementary document eTable 1. The protocol of this scoping review has also been successfully registered with before the completion of this project PROSPERO (# registration number: #1,340,319).

**Table 1:** The results of these NLP paper classification into different RA related clinical problems

NLP applications	Number of studies	Featuring articles
1. NLP studies involving0 RA related medication outcome, such as medication safety, utilization, indication, perception on comparative effectiveness and safety		
1a. Medication safety	2	[35, 36]
1b. Medication information, including medication use, indication	5	[37–41]
1c. Social media analysis for medication related perspective, safety, effectiveness	2	[42, 43]
2. NLP studies involving RA disease only, such as identifying RA disease only, RA phenotyping or RA related comorbidities or RA related disease activity		
2a. Feature enhancement for identification of RA diagnosis	4	[44–48]
2b. Feature enhancement for identification of RA phenotyping	6	[49–54]
2c. Data mining for identification of RA related comorbidities	3	[55–58]
2d. Feature enhancement for RA related disease activity, or severity	3	[59–61]

*NLP*  natural language processing, *RA* rheumatoid arthritis

### Identification

Our searches were conducted originally on Feb 27th 2026 from database inception, and recognizing the fast advancement of NLPs models in health informatics, we conducted an additional search on May 12th 2026. Our literature search was conducted as per following the guidance from the recommendations on comprehensive searches for systematic reviews [31–33]. We searched for the following keywords: “Natural language processing” + “Rheumatoid Arthritis” in key medical databases, including PubMed, EMBASE, Web of Science, and Directory of Open Access Journals (DOAJ) digital libraries. We selected these databases due to their comprehensive coverage of biomedical and clinical research. While computer engineering databases (e.g., IEEE Xplore, ACM Digital Library) may contain additional NLP studies, they were not considered because these databases usually covered studies that were more oriented towards algorithm development rather than clinical application. Given the focus of this review was on clinically applied NLP research in RA disease, specifically studies involving pharmacological and health outcomes, therefore, we only considered medical databases for systematic search.

**Fig. 1:**
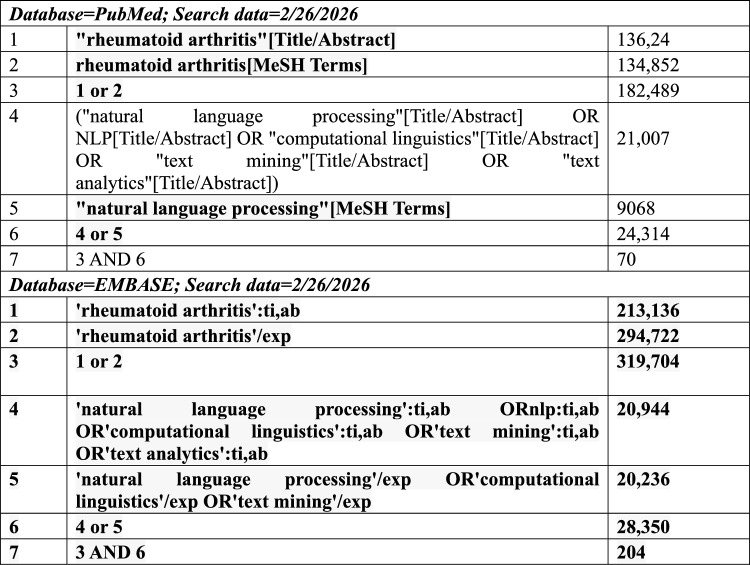
Search results

The search syntax related to NLP was developed and defined based on prior published NLP involved systematic reviews [22–25]. The literature search was conducted systematically considering free-text keywords as well as relevant MeSH ((Medical Subject Headings) terms. Additional MeSH terms included “Arthritis, Rheumatoid”, and “Natural Language Processing”. Of note, the keywords and relevant MeSH terms were considered in the searching process within the titles and abstracts of articles using Boolean operators, such as “AND,” “OR”, to enhance the comprehensiveness of the search strategy. Our search was limited English-language publications that incorporate any NLP method applied to medication-related interventions, because we hope to ensure the accurate interpretation and consistency in the following process of data extraction and analysis. We also manually screened the reference lists of eligible studies to additionally identify any potentially relevant articles. This scoping review primarily considered the selected databases (PUBMED and EMBASE), because of their broad coverage of biomedical, clinical, and health informatics, particularly pharmacy or medication relevant literature, in the interdisciplinary clinical studies involving rheumatoid arthritis and natural language processing research. Next, to manage the volume, these eligible citation records were then imported into Covidence platform to remove duplicates [34]. The full list of search syntax across each database, involving NLP terms and RA related MeSH/Emtree terms, is available in Fig. 1.

### Screening

In the screening, the titles and abstracts across each paper were reviewed, identified in the first step. We excluded any review, irrelevant ones, while retaining the full-text peer-reviewed papers accessible and relevant to our study. The review focused on full-text, English publication of clinical studies that involved any NLP technique and relevant to RA care. We excluded papers, which were focusing on persons irrelevance with RA. Additionally, there were some papers studied other AI based techniques, unrelated to NLP. We also removed papers that discuss RA care issues using non-AI approach or any duplicated reports.

To summarize, only original research articles were included, and review papers, conference proceedings, book chapters, etc. were excluded. We considered only papers focused on applying NLP ML technologies in addressing the clinical question related to the outcomes, safety, health and living quality of persons with RA or considering medication utilization, safety or effectiveness for persons with RA.

### Included

In the study inclusion steps, we thoroughly reviewed all eligible full articles that were included after the screening step to ensure the eligibility of all included studies. In the process of data extraction, the team focused on the type of clinical questions addressed, use of data source, use of NLP methods, and key findings. Each paper’s extraction was confirmed independently by two authors. If any disagreements occur, a team discussion was held, led by the leading author (YH) until a consensus is reached.

## Results

**Fig. 2:**
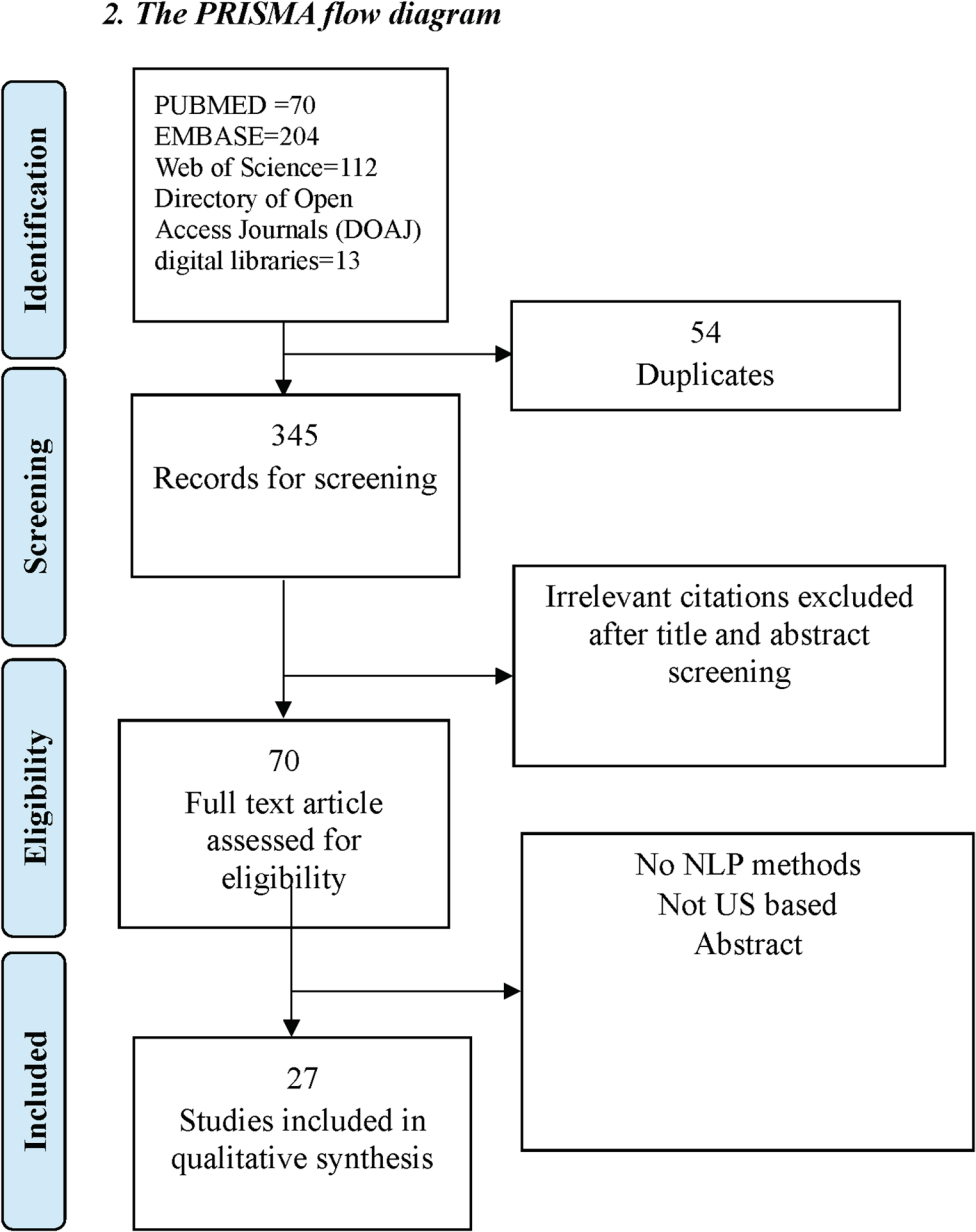
The PRISMA flow diagram

A total of 399 publication records were identified through the initial search terms (PubMed = 70, EMBASE = 204, Web of Science = 112, Directory of Open Access Journals = 13). After removing duplicates, 345 citations were considered for full-text article review. After assessing eligibility criteria, out of available title and abstract screening, 27 full-text studies met all inclusion criteria. From these, we thoroughly reviewed all 27 qualified publications. Table 1 shows the results of search terms. Figure 2 presents visually a PRISMA flow chart of the screening process of study selection.

### Characteristics of included studies

These 27 studies included were published between 2006 and 2026, with an observable increase in publication in more recent years. The study designs were mostly observational studies using real world datasets. Supplementary document eTable 3 shows the study characteristics of all included studies from this review.

### Use of NLP in RA care

The tasks involved in these eligible studies were divided into several categories to show how NLP was explored to advance RA care as follows. These NLP involved studies were broadly divided into medication related tasks or disease related tasks. Firstly, these NLP involved studies involved any medication related outcomes for patients with RA, including: (1) extract a variety of clinical features to support identifying medication safety or pharmacovigilance tasks (n = 2) [35, 36]; (2) extract information relevant to identification of medication use, medication mention, medication indication or other pharmacoepidemiologic characteristics, which were usually unavailable in structured component of datasets (n = 5) [37–41]; (3) social media analysis for identifying patients perspectives towards medication related perspective, safety, effectiveness (n = 2) [42, 43]. Secondly, these NLP involved studies focused on disease related tasks were described as following tasks: (1) aided in ICD diagnosis codes to improve the precise diagnosis of RA through extraction of information in the clinical notes (n = 4) [44–48]; (2) identification of RA patients with specific phenotypes (n = 6) [49–54]; (3) involved data mining for identification of RA related comorbidities (n = 3) [55–58]; (4) feature enhancement for identifying RA related disease activity, or severity (n = 3) [59–61]. Table 1 summarizes the categories of clinical tasks involved in these NLP based studies for RA care and corresponding featuring studies Table 2.

**Table 2:** The results of the NLP-involved paper classification into different data sources

Use of data sources for NLP applications	Examples	Featuring articles
Electronic Health Record (EHR) clinical documentation	Partners Healthcare network, Stanford Univ's EHR, unstructured clinical text within the Veterans Health Affairs (VHA) EHR system; EHR textual sources at University of California, San Francisco; Humedica electronic health records; EHR clincial narratives; EMR clinical narratives, chest computerized tomography reports	[35, 37–40, 44–61]
Pharmacovigilance and Safety Reports	FDA's Adverse Event Reporting System (AERS)	[36]
Public social media	Facebook, blogs, discussion boards, online posts	[42, 43]
Other medication focused online communities	RxNorm, SIDER, MedlinePlus	[41]

*NLP* natural language processing, *RA* rheumatoid arthritis, *EHR* electronic health records

### NLP techniques and data sources

Several primary sources of dataset used in these RA related NLP studies emerged were as follows. Specifically, the unstructured datasets leveraged using NLP were: data from electronic medical health records unstructured components [35, 37–40, 44–60], data from pharmacovigilance and safety reports [36], data from purely online social media texts [42, 43], or other medication focused online communities [41].

## Discussions

In this scoping review, we comprehensively examined multiple applications of NLP for RA related clinical studies to evaluate their added value and strong potential for informing clinical practice and health service research on addressing challenges encountered in the existing studies only relying on structured secondary datasets. Findings from this scoping review reflect a comprehensive and contemporary assessment of state-of-art research on NLP techniques in terms of improving RA care, highlighting dramatic research interests in NLP leveraged in RA related clinical studies, because we observe that eligible publications increasing in recent years. The studies included demonstrate exponential efforts and research interests in using NLP for RA, underscoring the expanding role and potential capabilities of NLP, particularly in RA related care, across a wide range of clinical tasks, such as strengthening the identification of RA diagnosis, RA related comorbidities, RA phenotyping and identification of RA related medication utilization.

### Comparison with other existing reviews on NLP use in general healthcare

While critical information related to a patient’s clinical functional status, progression of disease status and individual’s treatment response are available within a clinician’s qualitative documentation, much of such information remains inconsistently documented in the unstructured free-text EHR data in routine care, making it difficult to analyze. With NLP becoming an emerging tool that leverages AI to capture, understand and process semi-structured or unstructured textual data, many systematic reviews or original research studies have characterized the use of NLP to unstructured clinical data for cancer population, mental health, Alzheimer’s diseases, critical care, clinical trial prescreening eligibility, or general medicine [24, 62–73]. Overall, none has specifically evaluated the use of NLP to meaningfully assess the unmanageable clinical documentation of unstructured data for pharmacoepidemiologic and health outcome research for RA population. Other previous systematic reviews particularly relevant in RA considered general AI application, rather than specifically focusing on NLP for unstructured textual data [7, 27, 74]. As NLP techniques evolve rapidly, this scoping review adds to a body of reviews that focused on NLP tool assisted clinical tasks. For example, previous systematic reviews also reported a wide use of NLP in multiple clinical settings, including oncology, psychology, or Alzheimer’s diseases. Malgaroli et al. found that the NLP have gradually emerged as a tool in the field of study mental health related interventions, including evaluating patient symptoms, the risk of suicide, monitoring patients’ response to psychological treatment, as well as studying the provider and patient relational interactions [24]. Shakeri et al. systematically evaluated the use of NLP methods for Alzheimer’s disease analysis, and identifying that the NLP methods have been widely leveraged in detecting the Alzheimer’s diagnosis using speech datasets, identifying rich Alzheimer’s disease related factors using EHR’s unstructured components, as well as exploring the burden facing Alzheimer’s disease patients, and caregivers using social media datasets [62]. Sun et al. reviewed studies of NLP use that aided in the care of cancer patients, and found that NLP was used to improve the efficiency in cancer care by facilitating the information extraction and classification, which enables prevention, early detection of patient problems as well as improvement in personalized cancer related treatment decisions [63]. Our review, built upon the previous efforts adds to previous relevant publications by specifically examining the value of NLP leveraged in combining unstructured data to structured data in improving clinical care for RA. The various clinical tasks observed in our review aligns with these previous systematic reviews reporting the added value of NLP in facilitating various clinical tasks by extracting information from unstructured dataset. In particular, this analysis underscores the use of NLPs in enhancing the RA phenotyping, with around one fourth of studies focusing on NLP-driven phenotyping methods for RA, through explorations of additional clinical concepts extracted from physician notes, narrative text notes, manual chart review or billing codes data of hospital EHR/EMR [49–54]. The successfully application of NLP for EMR phenotype algorithms have also been previously specified else in other clinical problems, such as smoking and obesity [64]. For instance, Yang et al. integrated 2 rule-based NLP-derived phenotype tools in the population-level analyses for identifying smoking and obesity phenotyping, through evaluating a total of 19,215,303 unstructured clinical narrative notes of structured EHR fields covering 503,025 patients [64].

In this present review, other NLP-derived applications, notably using NLP to more precise identification of RA diagnosis [51–54], or capture RA related diseases activity measures [59–61] through analyzing clinical notes were also reviewed. Indeed, Yoshida et al. leveraged NLP to extract clinical concepts from physician-documented notes for 500 patients in addition to using EHR linking Medicare claims data in identifying gout flares, and found that such combined claims/NLP-concept model resulted in improvement in the identification of gout flares over existing claims-based algorithms [65]. Wang et al. used NLP to aid in physician chart review process, and further use such information to improve the efficiency of validating a claims-based algorithm for identifying intentional self-harm outcomes among obese patients from analyzing Medicare and Medicaid claims data [66]. Overall, these findings from this present review highlight the significant advancement of NLP applications in RA care in clinical practice.

### Comparison with current application of NLP in disease related practice

From this scoping review, one of the notable effects of NLP progress in RA is its advancement in the precise RA disease diagnosis and phenotyping. Bilgin et al. identified a meaningful number of AI applications in RA through analysis of structured datasets, including prediction of RA risk, assessment of RA related disease activity, RA related disease flares, prediction of treatment responses and assessment of comorbidities [74]. Though structured secondary data have been always favored by the clinical research community in RA, unstructured data powered with NLP and other text mining techniques gradually emerging as a new paradigm of powerful tool in the health service research field. The growth and wealth of large unstructured clinical free text powered with NLP advancement have brought beneficial impacts to advancing multiple areas of health service research, including healthcare care improvement, observational outcome research, medication safety, and broad development of clinical-based decision tool [75, 76]. Wang et al. have developed a NLP algorithm to extract cognitive scores from unstructured physician notes in EHR, and confirmed the NLP derived extracted cognitive function score yield high accuracy as compared to the CMS mandated cognitive data recorded in the Medicare-OASIS clinical assessment dataset [77]. Laurentiev et al. also developed a NLP tool to extract the status of activities of daily living (ADL) and instrumental activities of daily living (iADL) impairments through using the clinical notes containing 10,000 sentences of 441 people and 1000 sentences of 80 people [78]. In addition, for some clinical variables, such as treatment discontinuation or disease related phenotypes that are not routinely or commonly included in structured claims or EHR data, unstructured clinical notes may provide reliable source of such relevant pieces of information, thus making inclusion of clinical narrative notes important for accurate population-based clinical studies or pharmacoepidemiologic analyses. For example, in analyzing hospital EHRs of 36,656 patients receiving antipsychotics (APMs), benzodiazepines, warfarin, or oral anticoagulants, Yang et al. developed a NLP-based validation framework by evaluating the performance of an existing claims data-based treatment discontinuation algorithm against the medication discontinuation result determined from a NLP-aided chart review [79]. Liu et al. applied NLP to assist in the diagnosis of Alzheimer’s disease through analyzing the manually transcribed speech transcript samples for a total of 28 older adults, including 12 with AD and 16 cognitively healthy older adults [80].

### Comparison with current application of NLP in medication related practice

Moreover, another benefit of NLP lies in its ability to detect adverse events or signals of medication related adverse events through extraction of such information from physician’s unstructured textual data. While most of the EHR studies for assessment of medication safety are based on analysis of structured data through ICD coding system, one challenge is that the detection of adverse drug events (ADEs) is instead however often captured in the clinical narrative notes, rather than routinely recorded in the structured data field using diagnosis codes. NLP technique may provide an efficient and automated solution to the detection of ADEs from EHR notes and can be valuable for medication safety research. In the existing literature, a wide application in pharmacovigilance research have been observed for NLP-derived tools to extract clinical information to support medication safety surveillance [81, 82]. Sorbello et al. developed a NLP and other AI assisted tool to identify ADE signals from analyzing the free-text notes of Medical Information Mart for Intensive Care III (MIMIC III) EHR database’s discharge summaries to inform opioid medication safety [83]. Jagannatha et al. assessed the feasibility of NLP technologies to extract medication ADEs from analyzing 1089 annotated EHR notes from 21 patient sample with cancer at University of Massachusetts hospital, with diverse types of notes (discharge summaries, reports of consultation, and other notes taken in clinic) included [84]. In this present review, one study also proposed a NLP tool to screen potential ADEs for patients with RA through analyzing unstructured text information within FDA Adverse Event Reporting System (FAERS) combined with EMR data [33]. Notably, Geva et al. analyzed mentions of medications and relevant symptoms within clinical notes using NLP for pediatric population with pulmonary hypertension and found more comprehensive assessment of ADE rates identified from clinical notes over those purely ascertained using ICD diagnosis codes from administrative claims data [85]. Collectively, these studies underscore the capability and feasibility of NLP tools in summarizing and retrieving information through transforming unstructured text data into valuable insights of clinical ADEs, supporting drug safety research. In addition, another benefit of NLP associated with pharmacoepidemiologic and pharmacovigilance studies in RA is that NLP also advances medication-related outcomes research by improving our understanding of medication use, indications, and reasons for discontinuation, thereby bridging information gaps that are absent in structured datasets. Yusufov et al. used NLP to extract information on substance use by assessing a total of 9,821 chart review notes for 544 patients with heart failure [86]. In another retrospective cohort study involving 7009 diabetes patients, Wu et al. successfully applied NLP tool to extract reasons for insulin discontinuation [87]. Two other studies have also accurately extracted patients’ active medication use or the reasons for medication prescribed by using NLP tool based on information in progress notes or outpatient notes of patients’ charts [88, 89]. Furthermore, NLP tools can be valuable for extracting important clinical features from unstructured clinical text, and these features can supplement information obtained from structured EHR, hence, prediction models that incorporate NLP-derived features may provide greater predictive value than models that rely solely on structured EHR data. For instance, Haredasht et al. used NLP and large language model to incorporate 13 clinical and psychosocial features from unstructured free-text clinical note of Stanford’s EHR to predict treatment retention of buprenorphine-naloxone medications, and concluded prediction models incorporating NLP-derived features achieved improved model accuracy [90]. By analyzing EHR data of real-world advanced breast cancer patients with hormone receptor-positive (HR +)/HER2-negative characteristic in Spain hospital, Ribelles et al. also found that predictive model powered with NLP free-text processing features outperformed the baseline model solely in terms of predicting the progression to first-line treatment [91]. Taken together, the NLP systems can also be developed to extract, and encode information on adverse drug events, medication use or indications from clinical narrative reports of EHR systems.

### Clinical implications

Our review highlights the potential of NLP in enhancing RA clinical care and transforming clinical practice research for the population with RA, through addressing the challenges of aggregating clinical notes, discharge summaries and other unstructured real-world textual data. NLP, as an emerging tool uses AI to automatically process unstructured or semi-structured textual data, such as the RA related symptom assessment embedded in rheumatologist’s documentation [70]. As demonstrated in this review, many studies involved the development of multiple novel NLP models, not only those capable of improving the identification of RA diagnosis, but also those capturing important RA disease outcome [92]. Further, NLP can enable timely and consistent extraction of medication adverse events from clinical data, and identification of explicitly label mentions of reasons of RA medication prescription or discontinuation. Overall, findings from this present review suggest that NLP approaches holds potential for enhancing RA based clinical practice and research to address meaningful clinically relevant gaps embedded in clinician documentation. For example, the applications of NLP techniques learned from this scoping review may lay the foundation for future efforts in a large-scale comparative effectiveness and safety studies involving structured and unstructured data of combining administrative claims and electronic healthcare records data sources as NLP can automatically handle retrieval of information by transforming large, diverse, unstructured textual data into structured data format with scalability capability, such as assessment of RA-specific measure of symptoms status. This NLP powered approach has key clinical implications. First, this NLP tool can facilitate the initiatives related to RA-related quality measurement, as it allows a reliable avenue to track RA specific clinical assessment through transforming unstructured physician assessment into structured data. While EHR data contains structured, unstructured and semi-structured elements, > 80% information is contained in unstructured component. Such piece of information derived from the assistance of NLP tool can complement rheumatologist’s assessment and RA clinical documentation using the routine billing codes. For instance, England et al. used a validated NLP to obtain forced vital capacity values (FVC) from EHR notes, and later developed the RA-associated interstitial lung disease trajectory based on the NLP derived FVC value, enhancing the disease management and comorbidity monitoring [58].

Secondly, this NLP could also allow streamline and expedite the clinical trial recruitment for eligible RA patient identification, by facilitating efficient screening of large RA patient cohorts according to their disease severity status, and therefore accelerating the timely development of advanced therapies. For example, Cai et al. demonstrated the efficiency of improving RA clinical trial recruitment based on both the billing codes and NLP processed clinical variables [61]. Thirdly, this NLP model can also optimize treatment planning and support treatment decision-making, alerting patients and rheumatologists when medication safety signals, or informing downstream researchers about reasons for provider’s prescription indication or patients’ adjustment of treatment. Previous reviews also demonstrated the use of clinical NLP in improving the adverse drug event detection and pharmacovigilance [93–95]. Obviously, NLP tool enables the capture of information from clinical documentation complementing information not recorded in structured EHR.

### Challenges and future research directions

However, it is important to acknowledge the importance of validity of the output information derived from NLP models, which would be dependent upon a combination of factors, including (1) the practitioner’s input clinical notes, (2) the reliability of NLP model, (3) the management of these input entities for NLP analyses. As such, as researchers begin to leverage the NLP tool in the future, it could be a practical strategy to manually adjudicate advanced NLP analytics results with domain expert-based insights. To ensure broad use of reliable NLP methods for use cases across settings in RA practice, ensuring good agreement between NLP analytics and expert adjudication could be the first step. Next, the development and validation of framework of using unstructured data through NLP, to avoid bias from misclassification and improve internal validity in extraction of text entities may be considered as one necessary step to better support using NLP methods for more efficient and reliable collection of clinical outcomes in RA and the dissemination and evaluation of the methodology at other sites.

Overall, significant advances in NLP have reflected how we addressed clinical tasks by analyzing datasets involving free text language. The successes of NLP methods applied in RA care in extracting the clinical notes suggest the potential for NLP to transform the methodologic in the health service research and pharmacologic research in studying treatment related outcome, or RA related diagnosis, phenotyping and other disease outcome measures. While many innovations in the NLP methods for rheumatology have gradually emerged recently, several practical challenges still remain, before unleashing the full impact and great potential derived from such technological advancement. We further present a list of many challenges facing the interdisciplinary application of NLP in RA care and suggest multiple avenues to tackle them. Some notable topics include: (1) issue of data privacy, (2) data quality, (3) efficient, reliable, and standardized collection of data, (4) data interoperability, (4) appropriate dissemination of NLP derived results, (5) evaluation of the NLP methods at other data environment [96, 97]. Furthermore, to facilitate successful implementation, additional work is needed to construct population-representative, unbiased and sufficiently large datasets for RA care and to carefully select appropriate NLP approaches for RA research. Overall, the findings from this review enhance our understanding of the ability of NLP to digest large amounts of unmanageable clinical EHR unstructured text data into structured elements with meaningful clinical insights, which not only provides guidance for evidence-based practice in RA for rheumatologists but also highlights important implications for researchers interested in rheumatology.

Overall, in this scoping review, growing evidence showed that there observed substantial opportunities in the application of novel NLPs algorithms to synthesize, or analyze qualitative data collected from patients with RA, specifically focusing on pharmacoepidemiologic and health outcome research domains. While clinical NLP has contributed much progress recently in RA research, pragmatic challenges remain, including infrastructure needs, computational requirements and integration of data sources [98, 99].

### Strengths and limitations

This scoping review has several strengths. First, it offers a comprehensive summary of how NLP techniques are used across diverse clinical tasks related to RA care. This scoping review addresses an important gap in the existing literature and establishes a methodologic foundation for future investigations in leveraging unstructured datasets through the use of NLP for improving RA care. Second, this scoping review is considered to be one of the largest investigations on use of NLP technique applied to RA based clinical studies and research, and is one among the few that specifically focus on the NLP approaches in RA. Third, this scoping review broadly considers to include both retrospective real-world observational data, prospective experimental interventional studies and randomized controlled experimental data across various geographic areas in the US and globally and covering diverse populations, findings might be applicable to a diverse group with RA, with enhanced generalizability across health systems.

Nevertheless, there are also limitations to this proposed review that should be acknowledged. First, this review focused on the NLP approaches used for RA care and did not restrict to a particular and precise clinical task. We acknowledge that because of heterogeneity of study designs, patients’ characteristics, and NLP techniques, it did not allow direct comparisons and meta-analyses across studies included. Future researcher interested in conducting a meta-analyses might consider to define a more consistent in clinical task. Also, future research adopting more precise clinical question may consider more rigorous comparative evaluations of NLP methods across studies to better comment on their clinical applicability, model performance, validation strategies, and robustness across studies Moreover, this scoping review aimed to provide a comprehensive summary of the latest NLP methods for RA care, we did not comment on the types of model parameters or performance metrics even within the same category of clinical task, and as a result, we could not evaluate and compare across studies based on their quantitative performance. While the intention of this study is to offer a broad and comprehensive snapshot of the state-of-the-art NLP methods used in the field of RA care, focusing on the main clinical tasks these NLP methods applied to, and the clinical data sources used, we believe that a more detailed and in-depth evaluation of NLP method applied to a specific RA related task could be the directions of future work. Thirdly, although studies included reported different types of NLP techniques for RA care, findings from this scoping review do not represent the entirety of NLP techniques applied in clinical studies, as we considered only the research publications in the scope of RA focused clinical studies. As such, we also expect that important advancements in NLP will come from clinical areas outside of the RA domain, such as cancer and mental health care that were not covered in this present review. Lastly, another limitation is that the study eligibility criteria was limited by pre-defined review protocol and only focusing on inclusion of full-text peer-reviewed articles while excluding some recent conference abstracts papers. In particular, this might have affected the identification of available types of NLP used in RA related clinical studies, as several abstracts that have been excluded involved NLP for RA.

Taken together, in this scoping review, we showcased various NLP applications in RA related clinical settings, highlighting the potential of NLP in revolutionizing clinical practice by improving efficiency, accuracy, and patient care in the field of rheumatology. Nevertheless, future researcher might focus on: (i) extending the use of NLP into other range of clinical applications and practice settings; (ii) investigating the NLP tool’s scalability and generalizability to further advancements in the field of RA care; and (iii) further exploring the potential of other AI tools, such as transformer-based models (GPT-3 and BERT) utilized in clinical practice. With NLP continuing to make advancement, its impact of this AI tool on clinical practice is expected to increase, towards driving improvement in patient outcomes and efficiency in health systems.

## Conclusions

In summary, in this scoping review, we conducted a systematic summary of NLP methods for RA, focusing on clinical problems, datasets and NLP methods in detail. This scoping review found that the growing role of NLPs in a variety of aspects of RA, including RA diagnosis detection, identification of important RA related features and outcomes, text mining of the social media data, RA related phenotyping and others. While there are diverse applications, critical challenges remain, including standardized evaluation frameworks, ethical considerations and enhancing model interpretability NLPs can drive significant improvements in RA, through well integration of advancements in technology with clinical needs.

## Supplementary Information

Below is the link to the electronic supplementary material.

Supplementary file1 (DOCX 76 KB)

## Data Availability

The corresponding author can provide the material used and data analyzed on request.
