# Supplementary material for: Natural language processing to enhance rheumatoid arthritis care in clinical studies: a scoping review of applications, data, approaches, challenges and future directions
Source: Rheumatol Int. 2026 Jun 22;46(7):164. doi: 10.1007/s00296-026-06195-0 (PMC13287127; doi:10.1007/s00296-026-06195-0)
Supplement: Supplementary file 1 — Supplementary file1 (DOCX 76 KB) [file 296_2026_6195_MOESM1_ESM.docx]

**Electronic Supplementary Document [ESD] eTable 1. Study protocol involving inclusion/exclusion criteria for screening articles**

| *Population* | Population diagnosed with rheumatoid arthritis |
| --- | --- |
| *Intervention* | None |
| *Comparator* | None |
| *Outcomes* | Disease outcomes, or treatment related outcomes |
| *Study design* | Requiring real-world observational datasets |
| *Timing* | From database inception until Feb 27^th^ 2026 |
| *Setting* | US-setting |
| *Inclusion Criteria* | (1) Must use one natural language processing (NLP) method; (2) Must have evaluated patients with RA; (3) Must focus on analyzing treatment outcome or disease outcome for patients diagnosed for rheumatoid arthritis; (5) Be an original research paper; (6) Full text in English ; (7) US setting |
| *Exclusion Criteria* | (1) No NLP based modeling; (2) Not involving any population-level unstructured textual data; (3) Not focusing on RA patients; (4) Not US setting; (5) Not original research paper, e.g., review article |

**eTable 2. PRISMA-ScR Checklist**

| **SECTION** | **ITEM** | **PRISMA-ScR CHECKLIST ITEM** | **REPORTED ON PAGE #** |
| --- | --- | --- | --- |
| **TITLE** | | | |
| Title | 1 | Identify the report as a scoping review. | PAGE #1 |
| **ABSTRACT** | | | |
| Structured summary | 2 | Provide a structured summary that includes (as applicable): background, objectives, eligibility criteria, sources of evidence, charting methods, results, and conclusions that relate to the review questions and objectives. | PAGE #2-3 |
| **INTRODUCTION** | | | |
| Rationale | 3 | Describe the rationale for the review in the context of what is already known. Explain why the review questions/objectives lend themselves to a scoping review approach. | PAGE #4-5 |
| Objectives | 4 | Provide an explicit statement of the questions and objectives being addressed with reference to their key elements (e.g., population or participants, concepts, and context) or other relevant key elements used to conceptualize the review questions and/or objectives. | PAGE #6-7 |
| **METHODS** | | | |
| Protocol and registration | 5 | Indicate whether a review protocol exists; state if and where it can be accessed (e.g., a Web address); and if available, provide registration information, including the registration number. | PAGE #8 |
| Eligibility criteria | 6 | Specify characteristics of the sources of evidence used as eligibility criteria (e.g., years considered, language, and publication status), and provide a rationale. | PAGE #8 |
| Information sources* | 7 | Describe all information sources in the search (e.g., databases with dates of coverage and contact with authors to identify additional sources), as well as the date the most recent search was executed. | PAGE #8 |
| Search | 8 | Present the full electronic search strategy for at least 1 database, including any limits used, such that it could be repeated. | PAGE #8 |
| Selection of sources of evidence† | 9 | State the process for selecting sources of evidence (i.e., screening and eligibility) included in the scoping review. | PAGE #8-9 |
| Data charting process‡ | 10 | Describe the methods of charting data from the included sources of evidence (e.g., calibrated forms or forms that have been tested by the team before their use, and whether data charting was done independently or in duplicate) and any processes for obtaining and confirming data from investigators. | PAGE #9 |
| Data items | 11 | List and define all variables for which data were sought and any assumptions and simplifications made. | PAGE #9 |
| Critical appraisal of individual sources of evidence§ | 12 | If done, provide a rationale for conducting a critical appraisal of included sources of evidence; describe the methods used and how this information was used in any data synthesis (if appropriate). | PAGE #9 |
| Synthesis of results | 13 | Describe the methods of handling and summarizing the data that were charted. | PAGE #9 |
| **RESULTS** | | | |
| Selection of sources of evidence | 14 | Give numbers of sources of evidence screened, assessed for eligibility, and included in the review, with reasons for exclusions at each stage, ideally using a flow diagram. | PAGE #9 |
| Characteristics of sources of evidence | 15 | For each source of evidence, present characteristics for which data were charted and provide the citations. | PAGE #10 |
| Critical appraisal within sources of evidence | 16 | If done, present data on critical appraisal of included sources of evidence (see item 12). | PAGE #10-11 |
| Results of individual sources of evidence | 17 | For each included source of evidence, present the relevant data that were charted that relate to the review questions and objectives. | PAGE #10-11 |
| Synthesis of results | 18 | Summarize and/or present the charting results as they relate to the review questions and objectives. | PAGE #10-11 |
| **DISCUSSION** | | | |
| Summary of evidence | 19 | Summarize the main results (including an overview of concepts, themes, and types of evidence available), link to the review questions and objectives, and consider the relevance to key groups. | PAGE #11-18 |
| Limitations | 20 | Discuss the limitations of the scoping review process. | PAGE #19-20 |
| Conclusions | 21 | Provide a general interpretation of the results with respect to the review questions and objectives, as well as potential implications and/or next steps. | PAGE #21-22 |
| **FUNDING** | | | |
| Funding | 22 | Describe sources of funding for the included sources of evidence, as well as sources of funding for the scoping review. Describe the role of the funders of the scoping review. | PAGE # |

**Electronic Supplementary Document [ESD] eTable 3. Details of Study characteristics**

| **Table 2 Data sources, NLP methods and ML performance and outcomes** | | | | | | | |
| --- | --- | --- | --- | --- | --- | --- | --- |
| Author | Title | Study aims | Clinical tasks | Data sources | Sample size | NLP approaches and algorithm development techniques | Findings related to NLP performance |
| **1. NLP studies involving RA related medication outcome, such as medication safety, utilization, indication, perception on comparative effectiveness and safety** | | | | | | | |
| ***1a. Medication safety*** | | | | | | | |
| Wang 2018 | Detecting pharmacovigilance signals combining | To screen potential ADEs from both FAERS and EMR by incorporating NLP techniques to process EMR | Screening ADEs from clinical notes | Combining FAERS and EMR (The EMR data consists of all patient records at Mayo Clinic Rochester campus with Minnesota research permission over a period of 20 years (January 1995– October 2017) | 3,420-4,0161 | In the data preparation step, we apply NLP techniques to extract drugs and clinical outcomes, i.e., problems from EMR. | Signals detected from EMR have considerably overlapped with signals detected from FAERS or ADE knowledge bases, implying the importance of EMR for pharmacovigilance. |
| Lin 2014 | Automatic identification of methotrexate-induced liver toxicity in patients with rheumatoid arthritis from the electronic medical record | improve the accuracy of mining structured and unstructured components of the electronic medical record ( EMR) by adding temporal features to automatically identify patients with rheumatoid arthritis (RA) with methotrexateinduced liver transaminase abnormalities. Materials | higher level processing of the clinical narrative content, includ ing detailed information about the medication, other potential toxicity factors, and temporal-causal indicators | Partners HealthCare, Research Patient Data Repository, in cluded detailed data with timestamps for diagnoses, medications, problem lists, laboratory tests, procedures, and clinical notes from 1992 to 2013, | 5,903 | We aimed to develop an automatic CASE/NON-CASE classifica tion algorithm using a combination of NLP and classification rules. The goal was to first build and test a series of machine learning baseline systems using several competing non temporal feature sets (tested with a 10-fold cross-validation approach) in the training set. The preferred model was ex tended with combinations of temporal features to evaluate the contribution of each feature in the training set. The best feature rich model was then applied to a test set from Partners and an independent test set from the Vanderbilt. | We present a methodology for mining the wealth of clinical data in EMRs to automatically identify a temporally sensitive phenotype—MTX-related liver toxicity among patients with RA using a novel cTAKES module, DocTimeRel. |
| ***1b. Medication information, including medication use, indication*** | | | | | | | |
| Wei 2013 | Development and evaluation of an ensemble resource linking medications to their indications | To create a computable MEDication Indication resource (MEDI) to support primary and secondary use of electronic medical records (EMRs). | We applied natural language processing and ontology relationships to extract indications for prescribable, single-ingredient medication concepts and all ingredient concepts as defined by RxNorm. | The four resources included: (1) RxNorm (downloaded on June 4, 2012); (2) SIDER 2 (released on March 16, 2012)—a public medication knowledge base targeting adverse drug reactions extracted from FDA drug labels; (3) MedlinePlus (http://www. nlm.nih.gov/medlineplus)—an NLM-maintained website that offers consumer health information for patients, families, and healthcare providers; and (4) Wikipedia—an online collabora tively edited encyclopedia. RxNorm and SIDER 2 maintain indication information within a formal table structure with structured (ie, coded) medication and indication information. | MEDI contains 3112 medications and 63 343 medication–indication pairs. Wikipedia was the largest resource, with 2608 medications and 34 911 pairs | To obtain medication indications from MedlinePlus, we firs retrieved the webpage for each medication through the MedlinePlus Application Programming Interface (API) using the medication’s RxCUI as the query input. The resulting Hypertext Markup Language (HTML) pages were parsed and stored as text files. MedlinePlus maintains a consistent document structure for its drug monographs, although the text within each section is free-text. We found that certain sections of MedlinePlus fre quently contained drug indication information. We limited our analysis of the MedlinePlus description to the sections ‘Why is  this medication prescribed’, ‘About your treatment’, and ‘Other   uses for this medicine’, thus ignoring sections such as ‘What side  effects can this medication cause’ and ‘Precautions’. We used the  KnowledgeMap Concept Indexer (KMCI) to parse the free-text to obtain all non-negated ‘disease and finding’ concepts, as ‘ mapped to UMLS concepts. KMCI is a general-purpose natural language processing (NLP) engine that maps free-text documents to UMLS concepts and includes negation detection through an 20 adaptation of the NegEx algorithm. KMCI has performed MetaMap21 favorably in comparison with for medical school curriculum documents and has been validated in a variety of clin ical and education contexts | MEDI is a freely available, computable medication indication resource that is more comprehensive than existing freely available resources. Because it utilized UMLS concepts and ICD9 codes, MEDI can be easily used in conjunction with billing codes or concepts extracted from free-text using NLP. |
| Falasinnu 2026 | Pharmacoepidemiologic characterization of cannabis use and symptomatology in rheumatology using natural language processing of electronic health record clinic notes | To (1) quantify trends in documented cannabis use, (2) identify primary motivations (especially pain relief) and (3) examine sociodemographic and disease-related disparities. | We applied natural language processing (NLP) to 2.6 million electronic health record notes from 5051 adults with ARDs seen at a tertiary health center. NLP classified cannabis documentation as current, past, or none and identified reasons (pain, sleep, anxiety, nausea, appetite). | Electronic health records (EHRs) contain rich clinical documentation, including unstructured data where cannabis use is often recorded | 5051 adults with ARDs | We applied natural language processing (NLP) to 2.6 million electronic health record notes from 5051 adults with ARDs seen at a tertiary health center. NLP classified cannabis documentation as current, past, or none and identified reasons (pain, sleep, anxiety, nausea, appetite). | This study demonstrates the feasibility of using natural language processing to extract real-world evidence on cannabis use in autoimmune rheumatic diseases |
| Nelson 2015 | The use of natural language processing of infusion notes to identify infusions outpatient | Gibofsky 2017+A7 | We developed the NLP software using rule-based regular expression methods for extracting the infused drug, dose, and unit of measurement from medical record text notes. | infusion notes for US veterans enrolled in the VARA registry from 1 January 2007 to 30 September 2011. Patient data, procedures, and text notes were ob tained from the CDW and accessed through the Veterans Informatics and Computing Infrastructure (VINCI). | The VARA registry contained 115 patients who received 2029 inflixima between 1 January 2007 and 30 September 2011 | Two methods were used to retrieve potential infusion notes for NLP processing. We firs used a global search of all medical text notes from the patient cohort for the drug name  ‘infliximab’ brand name ‘ Remicaide®’, and any possible misspellings, abbrevi ations, or typos of those names. We also retrieved notes affiliate with a HCPCS code for inflixima infusion and notes close to the infusion visit date. These notes were linked using the unique visit ID from the procedure table and the text note metadata. | The use of NLP significantl improved the sensitivity and PPV for estimating infusion dates and doses, especially when combined with HCPCS codes. |
| Gibofsky 2017 | Effects of non-medical switching on outcomes among patients prescribed tumor necrosis factor inhibitors | To evaluate health care use and outcomes among patients who experienced a non-medical switch of their prescribed anti-tumor-necrosis-factor biological agent (anti-TNF) for cost containment reasons. | the impact of non medical switching on patients receiving anti-TNF biological therapy was evaluated using electronic medical record (EMR) data from the US to identify patients who switched anti-TNF biological agents for reasons related to cost and not due to side effects or efficacy concerns. | de-identified electronic medical record (EMR) data from Humedica, which was gathered from more than 20 medical provider organizations (medical groups, integrated delivery systems [networks of health care organizations under common ownership], hospitals) from around the US and covered the period from 2007 through 2013. | The NMS and control cohorts consisted of 158 patients and 4804 patients, respectively. | To evaluate the clinical impact of switching, subsequent physician notes were queried using NLP for documentation related to treatment “side effect“, lack of “efficacy“, or the combination of “side effect or lack of efficacy” within 30 days, 90 days, and 365 days following the index date. To assess the impact on prescribing, subsequent changes to another anti-TNF biological agent were identified within 30 days, 90 days, and 365 days of the index date. Whether this change was attributable to a physician-documented adverse clinical event was determined by using NLP to search for physician notes indicating “side effect” or dimin ished “efficacy” with the previous anti-TNF biological agent. | Non-medical switching among patients prescribed anti-TNFs was associated with increased health care use, medication-related side effects, and reports of diminished efficacy. |
| Garcia-Agundez 2024 | Structuring medication signeturs as a language regression task: comparison of zero- and few-shot GPT with fine tuned models | (1) examining the performance of LLMs to structure complex sigs, which can be framed as a language regression task that requires simple mathematical operations such as products, sums, and averages, (2) evaluating the mini mum number of manual annotations, provided as in-context examples, to achieve maximum performance, and (3) provid ing a comparison with a baseline consisting of smaller, fine ClinicalBERT tuned models, such as or BlueBERT. | To extract ing the average daily dose of 2 immunomodulating medications with frequent complex sigs: hydroxychloroquine, and prednisone using GPT-3.5 and GPT-4 with smaller fine-tuned models (ClinicalBERT, BlueBERT) | The data used in this study were derived from data aggre gated from 2 other observational studies examining medica tion dose. Briefly, one study was based at an academic medical center (UCSF Health) and involved all hydroxychlor oquine orders issued from the rheumatology or dermatology clinics for adult patients, between 2015 and 2020. Separately, a second study used data from Rheumatology Informatics System for Effectiveness, a national electronic health record (EHR)-based registry with data derived from practices17 over 300 and involved all oral glucocorticoid orders for patients with rheumatoid arthritis and Medicare insurance during 2018. | This yielded 702 hydroxychloroquine 200 mg sigs included in the current study, representing approximately 12 250 orders from 3000 patients.This yielded 22 104 sigs for predni sone or its equivalents included in the current study, repre senting approximately 194 500 orders from 44 500 patients. | However, significant model verbosity was observed, as the output would be contaminated by the model making up additional examples or adding undesired explana tions that would make the task of extracting the model’s numeric output from its textual reply another natural lan guage processing task in itself. This led us to discard this option. For prednisone sigs, as tab sizes vary, the tab size was included in the sig (eg, “4.0 mg tabs. take 1-3 tablets by mouth daily”). | Paired with minimal manual annotation, GPT-4 achieved excellent performance for language regression of complex medication sigs and vastly outperforms GPT-3.5, ClinicalBERT, and BlueBERT. However, the number of in-context examples needed to reach maximum performance was similar to GPT-3.5. |
| ***1c. Social media analysis for medication related perspective, safety, effectiveness*** | | | | | | | |
| Curtis | Social media for arthritis-related comparative effectiveness and safety research and the impact of direct-to consumer advertising | To (1) de scriptively characterize the demographics of people using social media to discuss rheumatoid arthritis (RA) and psoriatic arthritis (PsA) and psoriasis; (2) to evaluate the suitability of social media as a data source for drug safety research, particularly for the study of recently licensed molecules, and (3) classify the content and timing of the posts that these social media users are contributing, with a particular focus on communication related to newer biologic drugs and small molecules in relation to DTC advertising launch dates | (1) Analysis of pre-specified and empirically identified health related events and concerns associated with specific RA medications; (2) Analysis of Twitter data related to arthritis medication safety and tolerability | The Treato platform was used to analyze all publicly available social media data including Facebook, blogs, and discussion boards for posts mentioning inflammatory arthritis (e.g. rheumatoid, psoriatic). | 786,656 arthritis related posts | Over 2.5 billion posts were analyzed from these sources. Natural language processing (NLP) algorithms analyze this content to identify medical concepts mentioned in text and extract patients’ self reported descriptions of their health conditions and medica tions. More specifically, Treato takes medical terms and maps them to formal concepts in a medical ontology [6]. This process includes resolving conceptual synonyms of medical terms (e.g., myocardial infarction and heart attack); resolution of patient-specific terms (e.g., “ “pain in my joints” and “ “my joints hurt”) to medical terms; word-sense disam biguation algorithms (e.g., “ “BP” could refer to bi-polar disorder, blood pressure, or a bisphosphonate medication); and medication synonyms (e.g., generic and brand names for the same medication). Following the textual processing, posts are tagged with concepts, rather than just keywords, which allows for finer search and filtering capabilities. For example, a search for the herpes zoster concept would find posts referring to either  “herpes zoster” or  “shingles” (syno nym), but not to posts referring to  “shingles vaccine” (unless these posts also refer to “ “shingles” separately). In addition, the data are analyzed to extract patients’ specific experiences, making a distinction between posts that merely mention a disease and posts in which patients report having the disease. Other examples include identifying patients switching from drug A to drug B, and drug side-effect reports. Individual posts can be manually reviewed and classified by a subject matter expert. | The NLP classifier had a positive predictive value of 91% to identify HZ. |
| Xin | Fatigue, Pain, and Medication: Mining Online Posts Regarding Rheumatoid Arthritis From Reddit | To apply structural topic model to online RA discussions to identify and interpret the underlying topics | To provide a comprehensive understanding of the common concerns, symptoms, and feelings of RA patients in an online community, as well as analyze the impact of the COVID-19 pandemic on the topics of online discussions. | The online health discussion data come from Reddit, an American social media website for online users to post contents to share information and discuss topics in their communities | we extracted the submissions and comments from the subreddit r/rheumatoid, a subreddit that was created in March 2012 and is an active community with over 19,800 users | We performed an in-scope selection of the number of topics K. Specifically, our approach assessed two key criteria: semantic coherence and exclusivity. Semantic coherence is to indicate how often the words that belong to one topic co-occur in existing documents, and exclusivity is to indicate how exclusive the words can occur in a topic with high probability. | first, topics related to the treatment, medications, and symptoms discussion, including long-term medications, first-time medications, lab tests, appointments, pain1, pain2, and long-term symptoms have relatively higher topic proportions. This indicates that many RA patients have deep concerns about their symptoms and health conditions. Second, based on the topic prevalence difference before and after the COVID-19 pandemic, topic long-term symptoms and topic long-term medications became more prevalent after the pandemic |
| **2. NLP studies involving RA disease only, such as identifying RA disease only, RA phenotyping or RA related comorbidities or RA related disease activity** | | | | | | | |
| ***2a. Feature enhancement for identification of RA diagnosis*** | | | | | | | |
| Kronzer | Investigating the impact of disease and health record duration on the eMERGE algorithm for rheumatoid arthritis | to determine the dependence of the Electronic Medical Records and Genomics (eMERGE) rheumatoid arthritis (RA) algorithm on both RA and electronic health record (EHR) duration | How a RA algorithm correctly dientifies RA caese, according to HER duration and RA duration. | This population-based cohort study used the subset of Mayo Clinic Biobank participants who were also included in the Rochester Epidemiology Project (REP) or approximately 14 000 individuals. | we identified 497 patients with at least 1 RA diagnosis code. | The eMERGE RA algorithm was obtained from the eMERGE website. | eMERGE algorithm performance improves with longer RA duration as well as EHR duration up to 10 years, though shorter EHR lookback can improve identification of recently diagnosed RA cases |
| Carroll 2012 | Portability of an algorithm to identify rheumatoid arthritis in electronic health records | This study evaluated the portability of a published phenotype algorithm to identify rheumatoid arthritis (RA) patients from EHR records at three institutions with different EHR systems | Validating a phenotype algorithm | A database was created using Vanderbilt University Medical Center’s Synthetic Derivative, a de-identified copy of the EHR system (from the Partners Healthcare EHR utilized by Brigham and Women’s Hospital and Massachusetts General Hospital). Synthetic Derivative records are linked to DNA samples obtained from blood left over after routine clinical testing. | Partners ( n=500)Northwestern (n=390) Vanderbilt (n=376) | NLP system was used to search for the following: (disease concepts, laboratory results, medications, erosions | These results show that a previously published algorithm for RA is portable to two external hospitals using different EHR systems, different NLP systems, and different target NLP vocabularies |
| Liao 2010 | Electronic medical records for discovery research in rheumatoid arthritis | We assessed whether a classification algorithm incorporating narrative EMR data (typed physician notes), more accurately classifies subjects with rheumatoid arthritis (RA) compared to an algorithm using codified EMR data alone. | Whether NLP derived features strengthen the validity of a RA algorithm as compared to EMR based RA algorithm | We studied the Partners HealthCare EMR, which is utilized by two large hospitals, Brigham and Women's Hospital (BWH) and Massachusetts General Hospital (MGH), that combined, care for approximately 4 million patients in the Boston metropolitan area (Massachusetts, USA). The EMR began on October 1, 1996 for BWH and October 3, 1994 for MGH. | Subjects with =1 ICD9 RA code (714.xx) or who had anti-CCP checked in the EMR of two large academic centers were included into an ‘RA Mart’ (n=29,432). | We used five types of notes to extract information from narrative data: health care provider notes, radiology reports, pathology reports, discharge summaries, and operative reports. We utilized natural language processing (NLP) to extract clinical variables from the narrative data entered in a typed format (no scanned hand-written notes were used). We used the Health Information Text Extraction (HITex) system to extract the clinical information from narrative text. HITEx is an open source NLP tool written in Java and built on the General Architecture for Text Engineering (GATE) framework. The NLP application determines the structure of unstructured text records and outputs an annotated document tagging variables of interest (further details provided in Zeng et al., 2006. | We demonstrate the ability to utilize complete EMR data to define an RA cohort with a PPV of 94%, which was superior to an algorithm using codified data alone. |
| Huang 2020 | Impact of ICD10 and secular changes on electronic medical record rheumatoid arthritis algorithms | to compare the performance of an RA algorithm developed and trained in 2010 utilizing natural language processing and machine learning, using updated data containing ICD10, new RA treatments, and a new electronic medical records (EMR) system | We identified 53 144 subjects with at least 1 RA ICD code and at least 2 visit notes. | We used EMR data from two large academic hospitals in Boston, MA: the Brigham and Women’s Hospital (BWH) and Massachusetts General Hospital (MGH). BWH and MGH used the same locally-developed EMR system; the EMR was initiated at BWH on 1 October 1996 and at MGH on 3 October 1994. Epic EMR, a commercial EMR system was subsequently adopted in 2015 by BWH and 2016 by MGH | 16358 | We extracted narrative data using NLP from health care provider notes, radiology reports, pathology reports, dis charge summaries and operative reports. NLP was per formed using the Narrative Information Linear Extraction (NILE) package [26]. The NLP concepts extracted from the narrative data were: RA, psoriatic arthritis (PsA), SLE, seropositive, anti-CCP positive, erosions, metho trexate, anti-TNF, and all other DMARDs were included in a category called ‘other DMARDs’ (abatacept, ana kinra, azathioprine, cyclophosphamide, ciclosporin, hydroxychloroquine, gold, leflunomide, penicillamine, sarilumab, sulfasalazine, tocilizumab, tofacitinib, rituxi mab). The NLP mentions for each disease (RA, PsA, SLE) were summed; whereas medication and serology data were coded as never vs ever present | The 2010 RA algorithm validated with the updated data with similar performance characteristics as the 2010 data. While the 2010 algorithm continued to perform better than the rule-based approach, the PPV of the latter also remained stable over time |
| ***2b. Feature enhancement for identification of RA phenotyping*** | | | | | | | |
| Chen 2013 | Applying active learning to high-throughput phenotyping algorithms for electronic health records data | To assess the use of AL in identifying three phenotypes: RA, CRC, and VTE | The use of active learning (AL) in ML-based phenotyping algorithms | We investigated performance using two types of feature sets: unrefine features, which contained at least all clinical concepts extracted from notes and billing codes; and a smaller set of refined features selected by domain experts | 298 training samples | We investigated two different types of feature sets for each phenotype: unrefined features that included at least all billing codes (ICD-9, Current Procedural Terminology, etc.) and NLP-derived unified medical language system (UMLS) concept unique identifiers from clinical notes; and refined features, which included billing codes and UMLS concepts highly relevant to the specific phenotypes, as selected by domain experts. The NLP identifier3 tools included KnowledgeMap concept with ,20 38–40 41 SecTag MedLEE, and MedEx. | Our evaluation showed that AL outperformed PL on three phenotyping tasks. When unrefined features were used in the RA and CRC tasks, AL reduced the number of annotated samples required to achieve an area under the curve (AUC) score of 0.95 by 68% and 23%, respectively. AL also achieved a reduction of 68% for VTE with an optimal AUC of 0.70 using refine features. As expected, refined features improved the performance of phenotyping classifiers and required fewer annotated samples |
| Link 2022 | Binary acronym disambiguation in clinical notes from electronic health records with an application in computational phenotyping | To introduce a semi-supervised method for binary acronym disambiguation, the task of classifying a target sense for acronyms in the clinical EHR notes. | To develop a semi-supervised ensemble machine learning (CASEml) algorithm to automatically identify when an acronym means a target sense by leveraging semantic embeddings, visit-level text and billing information. | This is an observational study using clinical text and billing codes from the EHR of the Veterans Affairs Healthcare Centers Data for RA and MS were extracted from the Million Veterans Project [42] while data for MI were extracted from the general VA HER | 200 sample notes reviewed | We validated CASEml using note data from the Veteran Affairs (VA) to classify three acronyms: RA, MS, and MI. The results indicate that CASEml can accurately predict acronyms as well or better than state-of-the-art supervised methods in both sets of data. To further analyze the usefulness of CASEml, we evaluated the impact of applying CASEml to medical notes on a downstream NLP task: developing a phenotype algorithm for rheumatoid arthritis using EHR data. | we demonstrated that applying CASEml to medical notes improves the AUC of a phenotype algorithm for rheu matoid arthritis |
| Carroll | Naïve Electronic Health Record Phenotype Identification for Rheumatoid Arthritis | We used a cohort of physician-identified RA patients to evaluate the performance of a support vector machine (SVM) to accurately identify cases | Design of  phenotype identification algorithms | At Vanderbilt, a de-identified version of their EHR, called the ) Synthetic Derivative (SD)1, allows for privacy-preserving research. This has been used in conjunction with the Vanderbilt DNA biobank, BioVU, which accrues DNA samples from discarded blood samples | The cohort used in this analysis was a gold standard reviewed set of 376 individuals | We applied Support Vector Machines (SVMs) to both naïve (i.e., non-curated) and expert-defined collections of EHR features to identify Rheumatoid Arthritis cases using billing codes, medication exposures, and natural language processing-derived concepts | We show that with an expert defined feature set as few as 50-100 training samples are required. This study demonstrates that SVMs operating on non-curated sets of attributes can accurately identify cases from an HER |
| Liao 2015 | Development of phenotype algorithms using electronic medical records and incorporating natural language processing | to develop several phenotype algo rithms: depression,9 diabetes mellitus (V Kumar, in 9 preparation), inflammatory bowel disease (ulcerative colitis and Crohn’s disease),10 multiple sclerosis,11 and 10 ,11 rheumatoid arthritis. | To develop phenotype algorithm | EMR data | EMR at Partners Healthcare | NLP4 is a computational method for processing text to extract information using the rules of linguistics. When notes are processed, NLP breaks down sentences and phrases into words, and assigns each word a part of speech—for example, a noun or adjective. The NLP pro gram then applies the rules of linguistics to interpret the possible meaning of the sentence. In creating EMR phenotypes, we relied on the NLP task that identifie so-called concepts in narrative clinical text. A concept is a meaning; for example, the terms “atrial fibrilla tion( s)” and “auricular fibrillation(s)” are differen ways of expressing the same concept. | NLP improved all algo rithms using structured data by increasing the sensitiv ity while either maintaining or improving the accuracy, because NLP added independent predictive variables to the algorithm. |
| Wei 2015 | Combining billing codes, clinical notes, and medications from electronic health records provides superior phenotyping performance | evaluate the phenotyping performance of three major electronic health record (EHR) components: International Classification of Di (ICD) diagnosis codes, primary notes, and specific medications. Materials | Phenotyping performance | de-identified Vanderbilt EHR data. | 175 patients | Medication data in the SD are embedded in clinical narratives and were obtained with the MedEx NLP system in addition to electronic prescribing records from inpatient and outpatient order entry. MedEx extracts medication names and other signatures (dose, route, fre quency) from clinical narratives | EHR components provide a more consistent and higher performance than a single one for the selected phenotypes. We suggest cons idering multiple EHR components for future phenotyping design in order to obtain an ideal result. ....... |
| Kirby 2015 | PheKB: a catalog and workflow for creating electronic phenotype algorithms for transportability | report the current status and impact of the Phenotype KnowledgeBase (PheKB, http://phekb.org), an online environment supporti ng the workflow of building, sharing, and validating electronic phenotype algorithms. We analyze the most frequent components used in algorithms and their performance at authoring institutions and secondary implementation sites. Resul | Phenotyping algorithms | EHR data | 414 users | In addition to posting as sociated documents, researchers are encouraged to catalog their up loaded phenotype with metadata labels based on multidimensional representations, such as the methods and modalities used in the phe notype criteria (e.g., International Classification of Disease [ICD] codes, medications, natural language processing [NLP]), age, network, or in stitution affiliation. Uploaded documents typically include full descrip tions of the computable algorithms including data types used, execution logic and variable dependencies, data definitions, and flow charts or other descriptive graphics. | providing a central repository, PheKB enables improved development, transportability, and validity of algorithms for research-grade pheno using health care generated data. ..... |
| ***2c. Data mining for identification of RA related comorbidities*** | | | | | | | |
| Johnson 2025 | Risk and Temporal Trends of Heart Failure Subtypes in Rheumatoid Arthritis | Evaluate the risk and temporal trends of heart failure (HF) with preserved (HFpEF) and reduced ejection fraction (HFrEF) in rheumatoid arthritis (RA). | NLP supported to identify heart failure subtypes | Veterans Health Administration (VHA) administrative and health record data from 2000 to 2019 | We matched 67,850 patients with RA (mean age 62.5, 87.1% male) to 570,933 non-RA controls (mean age 61.1, 85.8% male). | Incident HF and HF-related death were queried, classifying HFpEF and HFrEF using left ventricular EF data from a validated natural language processing tool. | RA was most strongly associated with HFpEF and HFpEF-related death in this national-level, observational dataset. Heightened risks of HF subtypes have not improved despite advances in RA treatment. |
| Luedders 2023 | Enhancing the Identification of Rheumatoid Arthritis-Associated Interstitial Lung Disease Through Text Mining of Chest Computerized Tomography Reports | To evaluate if that including ILD-related terms identified within chest computed tomography (CT) reports through text mining would improve the PPV of these algorithms in this cross- sectional study. | ILD-related terms (e.g., ground glass, honeycomb) were identified in chest CT reports by natural language processing. | Using electronic health record data from a large academic medical center and performed medical record review to validate diagnoses | We identified 114 patients with =1 diagnostic or problem list code for both RA and ILD in the CRANE data warehouse | NLP was used to identify ILD-related terms (e.g., ground glass, honeycomb) in chest CT reports | The addition of ILD-related terms identified by text mining from chest CT reports led to improvements in the PPV of RA-ILD algorithms |
| Liao 2015 | Methods to Develop an Electronic Medical Record Phenotype Algorithm to Compare the Risk of Coronary Artery Disease across 3 Chronic Disease Cohorts | (1) to develop an algorithm that would enable the study of coronary artery disease (CAD) across diverse patient populations; (2) to study the impact of adding narrative data extracted using natural language processing (NLP) in the algorithm. | To develop NLP powered algorithm to identify CAD across RA and 2 other cohorts. | To create a list of potential structured data variables for a CAD pheno type algorithm, we obtained the ICD9 and CPT codes from the American Heart Association Get with The Guidelines—Coronary Artery Disease (AHA GTWTG-CAD)[13] (S1 Appendix). Narrative data extracted using NLP. Three board certified cardiologists (RM, PN, RS in Acknowledgements) created a CAD list of terms (customized dictionary) using descriptions corresponding to ICD9 codes from the AHA GTWTG-CAD (S1 Appendix). In addition, they provided terms they used to describe a patient with CAD in their clinical notes. These terms were mapped to concepts, e.g. coronary artery bypass graft (CABG). | We studied 3 established EMR based patient cohorts: diabetes mellitus (DM, n = 65,099), inflammatory bowel disease (IBD, n = 10,974), and rheumatoid arthritis (RA, n = 4,453) from two large academic centers | The concepts were extracted from narrative text notes using the Health Information Text Extraction (HITex) sys tem. HITex is an open source NLP tool which processes text notes and determines whether a note concept of interest was mentioned in the | We developed and validated a CAD algorithm that performed well across diverse patient populations. The addition of NLP into the CAD algorithm improved the sensitivity of the algo rithm, particularly in cohorts where the prevalence of CAD was low. |
| ***2d. Feature enhancement for RA related disease activity, or severity*** | | | | | | | |
| *Cheng 2025* | Inferring rheumatoid arthritis disease activity status from the electronic health records across health systems to enable real-world data studies | This study aimed to develop and validate scalable machine learning (ML) models to infer RA disease activity from EHR data. | Validating a RA related disease activity algorithm | EHR data from Mass General Brigham (MGB) and the Veterans Affairs (VA) | We studied 1105 MGB and 2631 VA RA patients | All clinical notes were processed using natural language processing (NLP) to identify mentions of RA and disease activity-related concepts from the narrative clinical notes. A dictionary of RA-related concepts was created through the Online Narrative and Codified feature Search Engine (ONCE) tool generated by the knowledge network mentioned above. | RA disease activity can be inferred at scale from within-institution EHR data, though cross-institution performance is limited. The inferred disease activity replicated association between RA and MACE and supports it's use in future studies to generate RWE. |
| *England 2023* | Extracting forced vital capacity from the electronic health record through natural language processing in rheumatoid arthritis-associated interstitial lung disease | To develop a natural language processing (NLP) tool to extract forced vital capacity (FVC) values from electronic health record (EHR) notes in patients with rheumatoid arthritis-interstitial lung disease (RA-ILD). | NLP to derivide a forced vital capacity (FVC) values from EHR notes | We performed this study within the Veterans Health Administration (VA) system, the largest integrated health care system in the United States. National administrative and EHR data stored in the VA Corporate Data Warehouse (CDW) was accessed within the VA Infor matics and Computing Infrastructure (VINCI) | We selected RA-ILD patients (n 7485) in the Veterans Health Adminis = tration (VA) between 2000 and 2020 using validated ICD-9/10 codes. | Clinical notes from October 1, 1999 to February 28, 2021 were obtained from the Text Integration Utilities (TIU) documents in the VA CDW. These are clinical notes entered into the EHR by providers (e.g., physicians) and staff (e.g., nurses, respiratory technologists). In addition to the content of the clinical notes, the date and location of the encounter for the corresponding clinical notes were collected, the latter using stop codes indicating VA specialty of care. Administrative records of PFT completion within the VA CDW were identified using Current Procedural Terminology (CPT) and ICD-Procedure Coding System (PCS) codes. | NLP of EHR notes increases the capture of accurate, longitudinal FVC values by three-fold over PFT equipment. Use of this NLP tool can facilitate pharma coepidemiologic research in RA-ILD and other lung diseases by capturing this critical measure of disease severity. |
| Cai 2021 | Improving the Efficiency of Clinical Trial Recruitment Using an Ensemble Machine Learning to Assist With Eligibility Screening | The objective of this study was to test whether an approach using electronic health record (EHR) data and an ensemble machine learning algorithm incorporating billing codes and data from clinical notes processed by natural language processing (NLP) can improve the efficiency of eligibility screening. | NLP features for clinical trial recruitment | EHR data were requested from the Partners HealthCare System Research Patient Data Registry, which contains comprehensive patient data, including structured data such as demographic information and billing codes for diagnosis, procedure, medication prescription, and laboratory test and unstructured clinical notes | In total, 3359 patients at Brigham and Women’s Hospital (BWH) and 642 patients at Faulkner Hospital (FH) were studied, with 461 (13.7%) eligible patients at BWH and 84 (13.4%) at FH. | To extract NLP concepts, we created a dictionary listing the concepts for each of the relevant recruitment criteria using the Unified Medical Language System (UMLS) (19). Specifically each dictionary contained a list of clinical terms and synonyms to represent the concept of each criteria item. Each concept was then mapped to a concept unique identifier in UMLS. For exam ple, based on the exclusion criteria “lymphoma diagnosis within 5 years,” we created a dictionary for the concept of “lymphoma,”, including the term “lymphoma” and all its synonyms existing in UMLS, such as “germinoblastoma”. We processed the clinical notes with a previously developed NLP tool, Narrative Information Linear Extraction (NILE) to obtain the number of times the concept is mentioned in the notes. NILE | The ensemble machine learning algorithm incorporating billing codes and NLP data increased the efficiency of eligibility screening by reducing the number of patients requiring chart review while not excluding eligible patients. |

**Electronic Supplementary Document [ESD] eTable 4 Description of NLP approaches**

| **NLP Application / Component** | **Conceptual Explanation ^[1-7]^** | **Example in Rheumatoid Arthritis Care** | **Featuring Studies** |
| --- | --- | --- | --- |
| **Text Preprocessing & Tokenization** | Preparing raw clinical text for analysis by cleaning, normalizing, and breaking it into meaningful units (words, phrases, or sentences), enabling downstream NLP tasks. | Identifying terms like “joint pain,” “RA flare,” or medication mentions in physician notes. | Chen et al., 2013; Wei et al., 2016; Carroll et al., 2012; Liao et al., 2010; Huang et al., 2020 |
| **Named Entity Recognition (NER)** | Automatically detecting and labeling key medical entities (diseases, symptoms, medications, lab values) from unstructured text, forming the basis for structured clinical data. | Detecting methotrexate, TNF inhibitors, fatigue, or joint swelling in EHRs. | Lin et al., 2015; Wei et al., 2013; Garcia-Agundez et al., 2024; Link et al., 2022 |
| **Information Extraction & Clinical Phenotyping** | Converting unstructured clinical text into structured data by extracting patterns, patient characteristics, or disease phenotypes, often combined with machine learning for cohort identification. | Identifying RA patients, RA-associated interstitial lung disease, or treatment patterns from EHRs and CT reports. | Carroll et al., 2011; Carroll et al., 2012; Liao et al., 2015; Luedders et al., 2023; Huang et al., 2020; Kronzer et al., 2020; Chen et al., 2013; Wei et al., 2016; Kirby et al., 2016; Liao et al., 2015 (PLoS One) |
| **Adverse Event & Safety Signal Detection** | Using NLP to automatically identify potential medication side effects, adverse events, or pharmacovigilance signals from clinical notes and EHRs. | Detecting methotrexate-induced liver toxicity or DMARD-related safety signals. | Wang et al., 2018; Lin et al., 2015; Falasinnu et al., 2026 |
| **Disease Activity & Outcome Inference** | Leveraging extracted text data to assess disease severity, progression, or patient outcomes, supporting monitoring and research in real-world settings. | Inferring RA disease activity status or evaluating treatment response from EHR notes. | Cheng et al., 2025; Liao et al., 2015; Johnson et al., 2025 |
| **Medication & Treatment Pattern Extraction** | Extracting detailed information about medications, dosages, treatment schedules, or therapy switches, enabling insights into treatment effectiveness and adherence. | Identifying DMARD prescriptions, infusion therapies, or TNF inhibitor switches. | Wei et al., 2013; Nelson et al., 2015; Garcia-Agundez et al., 2024; Gibofsky et al., 2017; Wei et al., 2016 |
| **Patient-Generated Text & Social Media Mining** | Analyzing text from patient forums, social media, or online health communities to capture patient-reported symptoms, experiences, or medication effects. | Mining Reddit or other online posts to study fatigue, pain, or medication experiences in RA patients. | Xin |
| References: (1). Wu S, Roberts K, Datta S, et al. Deep learning in clinical natural language processing: a methodical review.J Am Med Inform Assoc. 2020;27(3):457-470. doi:10.1093/jamia/ocz200; (2). Kreimeyer K, Foster M, Pandey A, et al. Natural language processing systems for capturing and standardizing unstructured clinical information: a systematic review.J Biomed Inform. 2017;73:14-29. doi:10.1016/j.jbi.2017. 07.012; (3). 2. Locke S, Bashall A, Al-Adely S, Moore J, Wilson A, Kitchen GB. Natural language processing in medicine: a review. Trends Anaesth Crit Care. 2021;38:4-9. doi:10.1016/j.tacc.2021.02.007; (4). Reading Turchioe M, Volodarskiy A, Pathak J, Wright DN, Tcheng JE, Slotwiner D. Systematic review of current natural language processing methods and applications in cardiology. Heart. 2022;108(12):909-916. doi:10.1136/heartjnl-2021-319769; (5). Lee RY﻿, Brumback LC﻿, Lober WB﻿, et al. Identifying goals of care conversations in the electronic health record using natural language processing and machine learning. (6). Lindvall C﻿, Lilley EJ﻿, Zupanc SN﻿, et al. Natural language processing to assess end-of-life quality indicators in cancer patients receiving palliative surgery. ﻿ J Palliat Med. 2019;22(2):183-187. doi:10.1089/jpm.2018.0326 (7). Prakash M Nadkarni, Lucila Ohno-Machado, Wendy W Chapman, Natural language processing: an introduction, Journal of the American Medical Informatics Association, Volume 18, Issue 5, September 2011, Pages 544–551, https://doi.org/10.1136/amiajnl-2011-000464 | | | |
